# A New Classification for the Relationship between Periodontal, Periapical, and Peri-implant Complications

**Published:** 2013-08-01

**Authors:** Mahdi Kadkhodazadeh, Reza Amid

**Affiliations:** a Department of Periodontics, Dental School, Shahid Beheshti University of Medical Sciences, Tehran, Iran; b Dental Research Center, Research Institute of Dental Sciences, Shahid Beheshti University of Medical Sciences, Tehran, Iran

**Keywords:** Classification, Peri-implantitis, Periodontal disease, Periapical Diseases

## Abstract

**Introduction:**

There are numerous studies supporting the high success rate of dental implants used for reconstruction of missing teeth. However, complications like mucositis and peri-implantitis are increasingly reported. Placement of dental implants in partially edentulous patients is associated with the risk of peri-implant diseases, especially when an old or a new inflammatory lesion is present adjacent to the implant site. Although no consensus has been reached on the difference in prevalence of peri-implant mucositis and peri-implantitis between fully and partially edentulous patients, available data clearly show that the combination of periodontal lesion and peri-implantitis is a possible risk factor for further complications. Several classification systems have been suggested for determination of the severity of disease around dental implants. However, no classification has been proposed for combined biological complications around teeth and implants. This study reviews the possible pathologic communication routes between natural dentition and the implants installed adjacent to them. Furthermore, we introduce a new classification system for the peri-implant disease in association with natural teeth called “PIST”. This system was designed based on the origin of the defects in order to clarify the different pathological situations which can be detected around dental implant. Using this classification system can help improve diagnosis, comparison and subsequent selection of the best treatment option.

## Introduction

In spite of the reportedly high success rate of dental implants, many clinicians encounter various soft and hard tissue complications around the inserted implants [1]. The success rate of osseointegration is high and predictable, although it can be further compromised by several biological and technical complications, which occur in association with dental implants and implant restorations. Whilst biological complications comprise many type of peri-implant diseases, a large variety of technical complications exist that are related to the mechanical damage of the superstructures. According to a review, analysis of the long term results of in fixed implant restorations, complications or failures occurred in 39% of patients during a 5-year observation period [2]. It should be noted that implant reconstructions with biological or technical complications are at greater risk of recurrent problems or failures [3]. Similarly, patients who have experienced implant failures have a 30% increase risk of further failures [4].

These complex situations can become more prevalent when dental implants are placed in partially edentulous patients adjacent to their natural dentition. Thus, it is necessary to have a clear understanding of the relationship between periodontal and peri-implant tissues. Several researchers have demonstrated similarities in colonizing bacteria between failing implants and the surrounding teeth in the same mouth [5-7].

In peri-implant diseases of partially edentulous patients, oral microorganisms can easily colonize the surface of teeth or dental implants and form oral biofilm. Microbial invasion induces inflammatory reactions in soft and hard tissues. Inflammation of the gingiva is termed gingivitis, while periodontitis, a more extensive/invasive condition also includes loss of supporting tissues around natural teeth. The corresponding conditions around implants are called peri-implant mucositis and peri-implantitis [8]. It is well- documented that infection around successful osseointegrated dental implants is the result of an imbalance between the bacterial flora and the host response. Peri-implant diseases may not only affect the peri-implant soft tissue (mucositis) but also involve the supporting bone (peri-implantitis) in crestal and/or apical areas [9].

In recent years, studies have focused on the biological aspects of peri-implant diseases occurring in partially edentulous individuals. They recognized that the composition of biofilm formed on implant surfaces, corresponded closely to those obtained from teeth surrounded by healthy tissues. Therefore, it can be hypothesized that the microbiota presenting in the oral cavity, may have a substantial impact on biofilm formation over newly placed implants. There are some similarities as well as dissimilarities between peri-implant diseases and periodontal infections [10]. Some authors reported similarities between the composition of the microbiota around teeth and implants in the same subject [7]. Most studies have demonstrated that the composition of the subgingival microbiota associated with health and disease conditions is similar around the implants and teeth; although, there is emerging evidence that differences may be present in some of the peri-implant infections [11]. A recent published study evaluated microbial differences between peri-implantitis and periodontitis in the same subjects using 16S rRNA gene clone library analysis and real-time polymerase chain reaction. The authors reported that the biofilm in peri-implantitis had a more complex microbial composition when compared with periodontitis. Common periodontopathic bacteria had a low prevalence, and several bacteria were identified as candidate pathogens in peri-implantitis [12]. Furthermore, longitudinal studies have investigated the transmission of putative periodontal pathogens from the neighboring periodontium to implant sites. The same putative periodontal pathogens were identified in residual periodontal pockets colonizing the implants after 3 and 6 months. The transmission of bacteria from tooth to implant sites was confirmed in studies investigating the dynamics of colonization [7].

The available data is too limited to draw conclusions about the possible differences in prevalence of peri-implant mucositis and peri-implantitis between fully and partially edentulous patients [13]. However, available evidence highlights the importance of eliminating potential reservoirs of periodontal pathogens before implant placement. It has also been demonstrated that maintaining periodontal health is essential in partially dentate patients with implants [14].

### Peri-implantitis and periodontitis

Candidates for dental implant placement should be informed about its potential risks if they have a history of periodontitis. In spite of the conflicting results, there is a general consensus that patients with a history of periodontitis show greater peri-implant bone loss and higher prevalence of biological complications than those with a healthy periodontium [15-17]. In addition, patients susceptible to periodontitis appear to be more susceptible to peri-implantitis than those with no such history. Although no documented data is available on the underlying conditions that may be considered as risk factors, a degree of genetic polymorphisms may play an important role [18-20].

Due to some similarities reported in the pathogenesis of peri-implantitis and periodontitis, it would be reasonable to use the same diagnostic criteria for detection of peri-implantitis and monitoring the progression of disease over time [21]. It can be concluded that periodontitis and peri-implantitis are not fundamentally different from the perspectives of etiology, pathogenesis, risk assessment, diagnosis and treatment. Diagnosis of periodontal disease and evaluation of the outcomes of periodontal therapy are usually based on clinical parameters *i.e.* clinical attachment loss, probing pocket depth, and bleeding on probing. Nevertheless, some differences in the host response to these two infections may explain the occasional rapid progression of peri-implantitis [8, 11]. Consequently, a diagnosed peri-implantitis should be treated at an early stage without delay.

### Apical peri-implantitis and periodontitis

Endodontic lesions that remain in extraction sockets before immediate implantation or around the remaining dentition may be considered a major cause of peri-implant diseases [22]. Apical peri-implantitis is a commonly used term for every radiolucent lesion detected around the apical parts of implants. A periapical lesion around a dental implant called retrograde peri-implantitis is defined as a clinically symptomatic lesion at the apex of an implant, while the coronal portion of the implant is totally intact [23]. The clinical symptoms may include pain, tenderness, swelling and/or presence of a fistula. Our knowledge about the incidence, etiology, and treatment of periapical lesions around implants is scarce. Different etiologic factors have been suggested to play a role in the emergence of retrograde peri-implantitis such as excessive heating of bone during bone drilling [24], failed endodontic and/or apicoectomy procedures [25], placement of an implant in close proximity of an existing infection [26], excessive trauma during implant placement [27] and presence of pre-existing microbial pathology [28-30]. Data obtained by a retrospective analysis show that in case of endodontic treatment of a tooth or presence of a periapical lesion, the potential incidence of a periapical lesion around the implant replacing that tooth would be 8.2% to 13.6% (odd ratio=7.2). Periapical pathology of the adjacent teeth further raises this percentage to 25%. Irrespective of the etiology and the treatment plan, the authors found bacteria (mostly P. gingivalis) in nine lesions. They concluded that presence of an endodontic pathology in the extracted or neighboring teeth can significantly increase the likelihood of a periapical lesion developing around the future implant [31]. A brief conclusion about the apical peri-implantitis has recently been published by the authors [32].

### Classifications of peri-implant disease

Progression of peri-implant mucositis to peri-implantitis is gradual and the progression of peri-implantitis may be slow. Thus, exact determination of the onset of peri-implantitis is not feasible. Detection of cases at early stages of disease with high specificity is quite challenging as well. On the other hand, in case of slow and gradual progression of disease, it is extremely important to stop the infection early to prevent massive destruction of peri-implant tissues. Advanced peri-implantitis is rarely seen as it is usually intercepted at an earlier stage [33]. Thus, using a simple and sophisticated system to classify and differentiate the wide range of peri-implant diseases can be helpful for early detection and treatment of patients. Recently, a new scoring system named Implant Success Index (ISI) was presented by the authors to facilitate the classification of implant defects [34]. The published data confirmed the validity and popularity of ISI among clinicians; although, it has been designed only for peri-implant lesions. When dental implants are placed in partially edentulous patients, combined lesions involving both the implants and the natural remaining teeth make the situation more complicated. Therefore, the present study aimed to introduce a comprehensive system to classify the peri-implant and periodontal diseases that may be detected simultaneously (Table 1). The available systems were basically designed to show the clinical and radiographic findings of infection and tissue destruction around dental implants. In other word, they have no specific criteria to differentiate the original etiology of the disease in partial edentulous patients. Therefore, we are introducing a new classification system to make the clinical diagnosis and treatment planning easier through a more sophisticated route. This classification helps clinicians to manage peri-implant diseases easier because it provides a better understanding of the etiology.

**Table 1.**
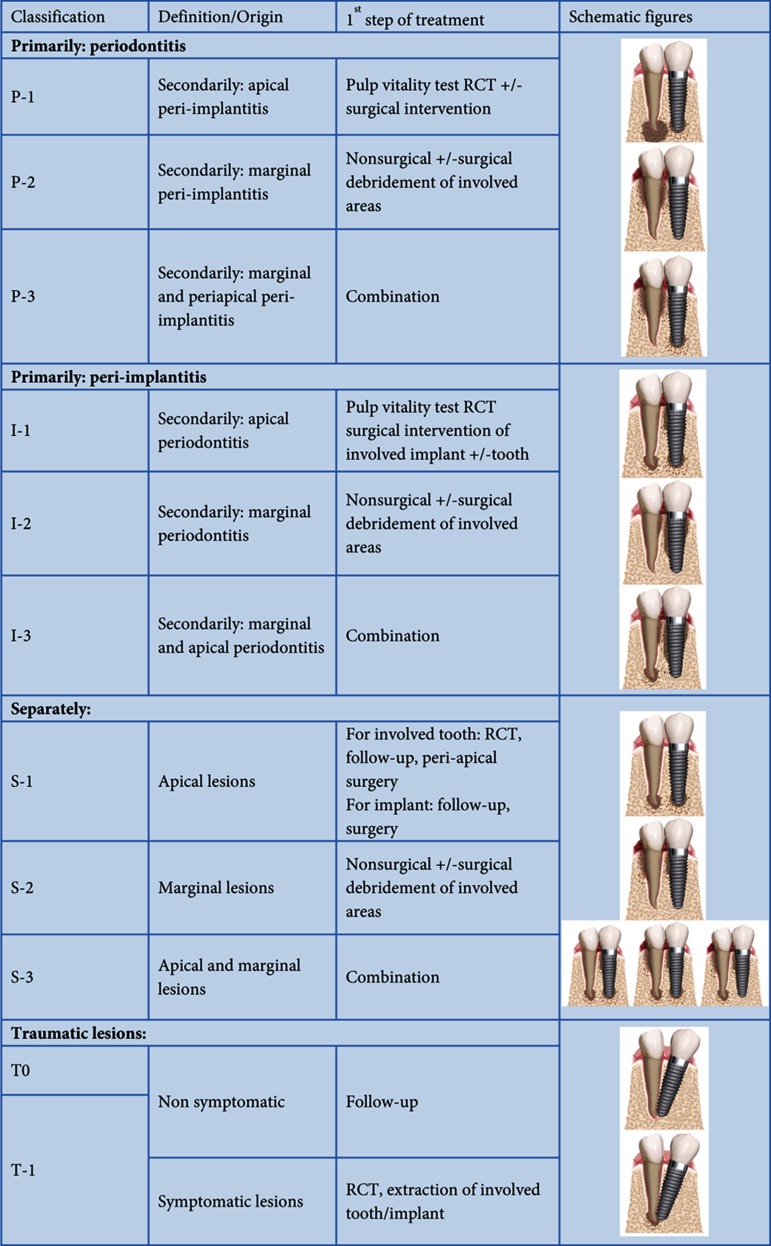
PIST Classification: A Method for Classification of Combined Peri-implant, Periodontal and Periapical Lesions

### PIST Classification System

*Primary periodontal lesions (P-class):* The origin of the combined lesions in this category is the periodontium of the remaining dentition. In situations like this, marginal or apical periodontal lesions involving the neighboring implant are a common finding. Based on the extension of the involved area around implant as a secondary defect, 3 different subgroups introduced: P1: apical, P2: marginal and P3: marginal and apical peri-implantitis. The access for tissue debridement and control of host response are easier around natural teeth than dental implants. Thus, it is not surprising that all clinicians insist on implantation in patients with periodontally healthy dentition. In a variety of situations, surgical access to the contaminated implant surface may be required and an open-flap procedure is carried out to access the affected surface. The typical saucer-shaped and deep peri-implantitis lesions can only be decontaminated effectively through a surgical access. While periodontal treatment involves the debridement of affected tooth surfaces, treatment of peri-implantitis focuses on the decontamination of implant surfaces.

*Primary Implant complications (I-class):* The combined lesions may origin from implant side due to improper plaque control and biological consequences. Like as P-class, three different subgroups based on the extension of involvement were clarified. Available data clearly show significant differences in histopathology and rate of disease progression between peri-implantitis and periodontitis [35]. As discussed earlier, the inability to access microbial habitats in the subgingival/submucosal region may often lead to less than optimal treatment outcomes. Irrespective of surface roughness and configuration, decontamination of the titanium surface poses inherent problems and can probably not be achieved by mechanical debridement alone. Irrigation with antiseptic and/or physiologic saline solutions may dilute the bacterial load, thereby allowing the innate and adaptive host responses to control the infection.

*Periodontal and peri-implant lesions that occur simultaneously but separately from each other (S-class): *The time interval between the development of lesions around natural teeth and implants clearly indicates that the two lesions have occurred separately. In other words, it is a simple coincidence rather than a true combination. There is no direct relationship between the periodontal and peri-implant diseases in this situation. Thus, the treatment options would be different for each lesion independent of one another. However, the peri-apical lesions generally have more complex outcomes. If both of the lesions were present around the apex of tooth and implant, it would be called S-1. S-2 represents two separate marginal defects. Several situations can be detected in S-3, when some variations of marginal and periapical lesions can be detected. Presence of periapical radiolucency affects the success rate. It seems that a higher success rate is achieved in teeth without periapical radiolucency as compared to those with a periapical lesion [36]. S-class lesions may be more complex and more difficult to manage. Some authors have stated that an evaluation period of 1-2 years is sufficient to observe the success rate in teeth without periapical periodontitis, while in the presence of periapical lesions, a period of 2-5 years may be required [36]. In addition, clinical symptoms usually resolve within several hours or days following the initiation of root canal therapy. However, complete healing of the periapical bony lesion may require several months or even years [37, 38]. Surgical treatment is a good alternative when nonsurgical retreatment is not successful, not indicated, or not feasible. Surgical retreatment removes the periapical lesion or other irritants from the periapical tissues, allows healing and it has the highest success when it is done with root-end preparation and a biocompatible root-end filling [39].

*Traumatic lesions with an iatrogenic origin that occur during implant placement next to a natural tooth (T-class): *Some problems that occur during implant installation, such as over-heating and over-instrumentation, are considered traumatic for the surrounding structures like PDL of neighboring teeth. In T1 cases, the involvement of a vital tooth is severe and symptomatic and root canal therapy may be indicated. In other asymptomatic lesions categorized as T0, informing the patient and radiographic follow up are recommended.

### Advantages of PIST Classification:

a. *Comprehensive:* it covers all possible combined lesions that may be seen.

b. *Easy to use:* it is simple, logical and reasonable.

c. *Acceptable:* it is not a strange system for many clinicians due to the same principles that are used for combined periodontic-endodontic lesions.

d. *Reasonable:* it has been mainly designed on understanding of the origin and location of lesion. Thus, we introduced treatment recommendations through an objective way.

e. *Wide use in clinic:* it will be helpful for determining the prognosis and treatment guidelines.

## Conclusion

Peri-implant diseases especially those in association with the neighboring teeth must be thoroughly evaluated. A systematic classification of the complications that occur around dental implants inserted in dentate jaws may fulfill the demand for a comprehensive decision-making process. Multiple factors must be evaluated individually and a thorough treatment planning is required.
